# Low-Abundant Microorganisms: The Human Microbiome’s Dark Matter, a Scoping Review

**DOI:** 10.3389/fcimb.2021.689197

**Published:** 2021-05-31

**Authors:** Jéssica Alves de Cena, Jianying Zhang, Dongmei Deng, Nailê Damé-Teixeira, Thuy Do

**Affiliations:** ^1^ Department of Dentistry, School of Health Sciences, University of Brasília, Brasilia, Brazil; ^2^ Department of Preventive Dentistry, Academic Center for Dentistry Amsterdam (ACTA), University of Amsterdam and VU University Amsterdam, Amsterdam, Netherlands; ^3^ Xiangya School of Stomatology, Xiangya Stomatological Hospital, Central South University, Changsha, China; ^4^ Division of Oral Biology, School of Dentistry, University of Leeds, Leeds, United Kingdom

**Keywords:** next-generation sequencing, human microbiome, low-abundant microrganisms, scoping review, minority microbiota

## Abstract

Research on the human microbiome has mainly been restricted to the identification of most abundant microbiota associated with health or disease. Their abundance may reflect their capacity to exploit their niche, however, metabolic functions exerted by low-abundant microrganisms can impact the dysbiotic signature of local microbial habitats. This scoping review aims to map the literature regarding the management of low-abundant microorganisms in studies investigating human microbiome samples. A systematic literature search was performed in 5 electronic databases, as well as grey literature. We selected clinical microbiome studies targeting human participants of any age, from any body site. We also included studies with secondary data which originated from human biofilm samples. All of the papers used next-generation sequencing (NGS) techniques in their methodology. A total of 826 manuscripts were retrieved, of which 42 were included in this review and 22 reported low-abundant bacteria (LB) in samples taken from 7 body sites (breast, gut, oral cavity, skin, stomach, upper respiratory tract (URT), and vagina). Four studies reported microbes at abundance levels between 5 and 20%, 8 studies reported between 1 and 5%, and 18 studies reported below 1%. Fifteen papers mentioned fungi and/or archaea, and from those only 4 (fungi) and 2 (archaea) produced data regarding the abundance of these domains. While most studies were directed towards describing the taxonomy, diversity and abundance of the highly abundant species, low-abundant species have largely been overlooked. Indeed, most studies select a cut-off value at <1% for low-abundant organisms to be excluded in their analyses. This practice may compromise the true diversity and influence of all members of the human microbiota. Despite their low abundance and signature in biofilms, they may generate important markers contributing to dysbiosis, in a sort of ‘butterfly effect’. A detailed snapshot of the physiological, biological mechanisms at play, including virulence determinants in the context of a dysbiotic community, may help better understand the health-disease transition.

## Introduction

Advances in high-throughput sequencing approaches have revolutionised microbiology and enabled the characterization of the complex ecological contents of microbial communities, however, our understanding of the mechanisms impacting host-microbial homeostasis remains limited (Hajishengallis et al., 2012). Changes to the human gut microbial composition, for example, can influence host health and diseases, and may affect the microbiota at other body sites (Banerjee et al., 2018). A concept of pathogenicity influenced by both microorganisms and the host has been proposed in the damage-response framework (Casadevall and Pirofski, 2003).

Research on the human microbiome has mainly been restricted to comparisons of the most abundant organisms and the identification of a “core” microbiota associated with health or disease. Indeed, the core microbiome may reflect their capacity to exploit their niche, being favoured by nutrients, O_2_ concentrations, etc. to allow surface colonisation. However, opportunistic pathogens may contribute to the compositional and or functional shift towards dysbiosis and could be among the minority taxa. Key species could therefore easily be overlooked in next generation sequencing (NGS) analyses (Turnbaugh et al., 2007; Zerón, 2014).

Furthermore, studies using a 16S rRNA metagenomic approach are limited to the identification of bacteria and archaeae (arguably accurately to the genus level), leaving the view of the richness and diversity of the whole microbiome incomplete and underestimated (Brooks et al., 2015). This is certainly true for *Methanobrevibacter smithii*, a member of the *Archaea* domain in a relatively minor constituent of the gut microbiome that contributes to bacterial metabolism in ways that promote host dysbiosis (Hajishengallis et al., 2012). This species and its methanogenic relatives, though in low abundance, have been demonstrated to be capable of providing conditions for the growth of pathogenic bacteria in periodontal sites, driving to periodontitis (Lepp et al., 2004). The composition of the microbial communities can be misinterpreted regarding the presence of virus, archaea, and fungi, making it a challenge to gain a holistic view.

Subsequently, low-abundant microrganisms could be considered the “dark matter” of the human microbiome. Recent studies (Hajishengallis and Lamont, 2016; Wang et al., 2017; Banerjee et al., 2018; Stobernack, 2019; Berg et al., 2020; Xiao et al., 2020) are paying more attention to these organisms, and increasingly taking into account the “keystone species” concept, corresponding to organisms which effect on the community is disproportionately large compared to their relative abundance (Power et al., 1996). A similar concept in macroecology suggests species in low abundance have a major role in their respective community (Hajishengallis et al., 2012). Abundance is the factor differentiating keystone microorganisms from those that are dominant. A dominant species might affect the environment exclusively by its sheer abundance, while a keystone microorganism may influence metabolic functions of the microbiome, despite its low abundance. Examples of keystone pathogens are: *Porphyromonas gingivalis* associated with periodontitis (Holt and Ebersole, 2005; Perez-Chaparro et al., 2014; Burmistrz et al., 2015; Camelo-Castillo et al., 2015; Ai et al., 2017; Stobernack, 2019), *Klebsiella pneumonia*, *Proteus mirabilis* (Garrett et al., 2010), and *Citrobacter rodentium* (Bry et al., 2006) associated with intestinal inflammatory diseases; and *Fusobacterium nucleatum* (Kostic et al., 2013; Rubinstein et al., 2013) associated with colon cancer (Banerjee et al., 2018). Furthermore, studies investigating *Bacteroides fragilis*, a pro-oncogenic bacterium, have found it to be a minor constituent of the colon microbiota in terms of relative abundance. Its unique virulence characteristics, such as secretion of a zinc-dependent metalloprotease toxin, alter colonic epithelial cells and mucosal immune function to promote oncogenic mucosal events, in which in addition to the intraluminal environment, enhance the oncogenic process. This gave rise to the concept of “alpha-bugs”, due to its ability to be directly pro-oncogenic but also to be capable of remodeling the entire healthy microbiota (Sears and Pardoll, 2011; Hajishengallis et al., 2012). Thus, the identification of low-abundant organisms within a microbial population associated with disease could be crucial. Unless we have a more “complete” view of the microbiota, including an accurate detection of low-abundant species, our understanding of the microbiology remains limited, as well as our strategy to improve therapy designs/interventions in diseases with polymicrobial cause.

Studies of the minority microrganisms may reveal unique signatures, which could lead to diseases. Hence, a much deeper characterization of their presence in the microbiome in which they are involved is desirable. This scoping review aims to map the literature regarding the management of low-abundant organisms in studies investigating human samples. We aimed to determine: 1) How researchers classify organisms as low-abundant; 2) How they handled and processed NGS data of low-abundant organisms bioinformatically and 3) The distibution of low-abundant microorganisms among various body sites.

## Methods

### Study Design

This is a scoping review to map the literature on low-abundant organisms in the human microbiome, conducted using the PRISMA Extension for Scoping Reviews (PRISMA-ScR) checklist (Tricco et al., 2018).

### Search Strategy

**Figure 1:**
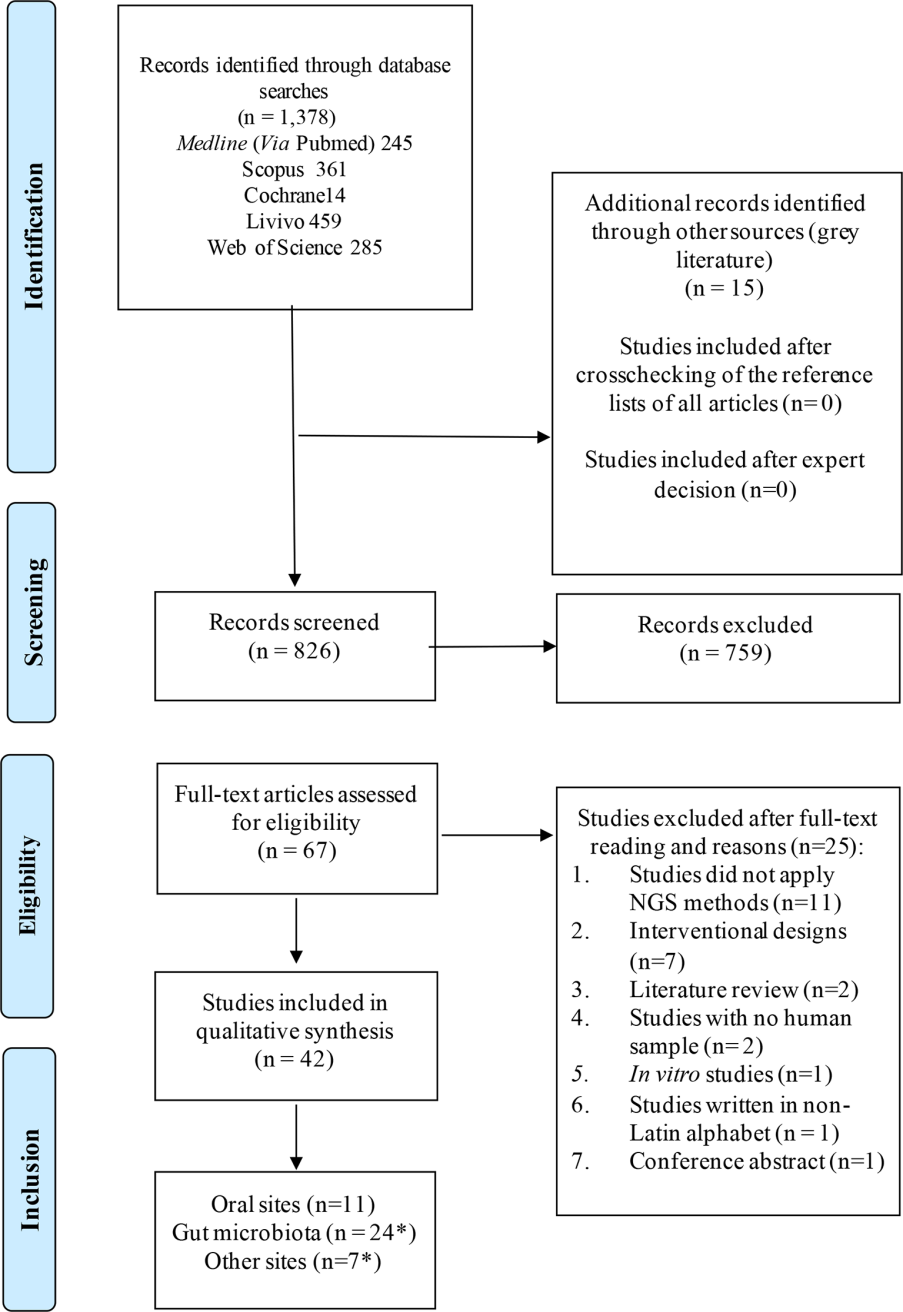
Flow diagram for study selection according to PRISMA guidelines. *Some studies sampled multiple sites in one study.

Systematic literature wide opened search was performed in electronic databases, also including the grey literature (**Figure 1**). General controlled vocabulary (MeSH Terms) and keywords were used and the searches had no language, year, or publication type restriction. The main terms included “microbiota”, “microbiome”, “human microbiota”, “low abundant”, “minority species”, “keystone”. The search strategy and the results retrieved in each electronic database are shown in **Appendix 1**. Duplicated references were removed by the reference manager EndNoteWeb (Clarivate Analytics, Mumbai) and then manually.

### Eligibility Criteria

Studies were included if they satisfied all the following criteria: (1) clinical studies where the target population consisted of humans of any age who were donors of samples from any site; (2) the study design was either a observational study, case series, or any other type of clinical study or studies with secondary data originated from humans; and (3) studies with any term related to low-abundant organisms (e.g. keystones, minority species) in title or abstract.

Studies were excluded if: 1) Studies did not apply next-generation sequencing (NGS) methods to evaluate the microbiota; 2) They were designed as intervention studies; 3) They were literature review, conference abstracts, *in vitro* or animal studies, or any other kind of study carried out without human samples in a primary or secondary analysis; and 4) They were written in a non-Latin alphabet.

### Selection of the Manuscripts

Two reviewers, JAC and JYZ, independently screened the eligibility of all identified titles and abstracts for inclusion in the full-text review at the Rayyan QCRI® (Qatar Computer Research Institute, Qatar). Any conflict that arose were resolved by a third reviewer. The same reviewers evaluated full-text articles for inclusion using the same inclusion and exclusion criteria. The list of selected articles was analysed to identify manuscripts that could have been lost during searches in the electronic database.

### Data Extraction

Data extraction was performed by the two reviewers independently, and included the following information: Author (year), country, design of the study, range of age of patients, sampling site, type of sample, the platform of sequencing; method of sequencing (16S rRNA or metagenomics or metranscriptomics), method of data analysis and bioinformatics; and abundance of species considered as low-abundant/minority microrganisms. All extracted data was checked by a third reviewer.

## Results and Discussion

### Characteristics of the Selected Studies

**Figure 2:**
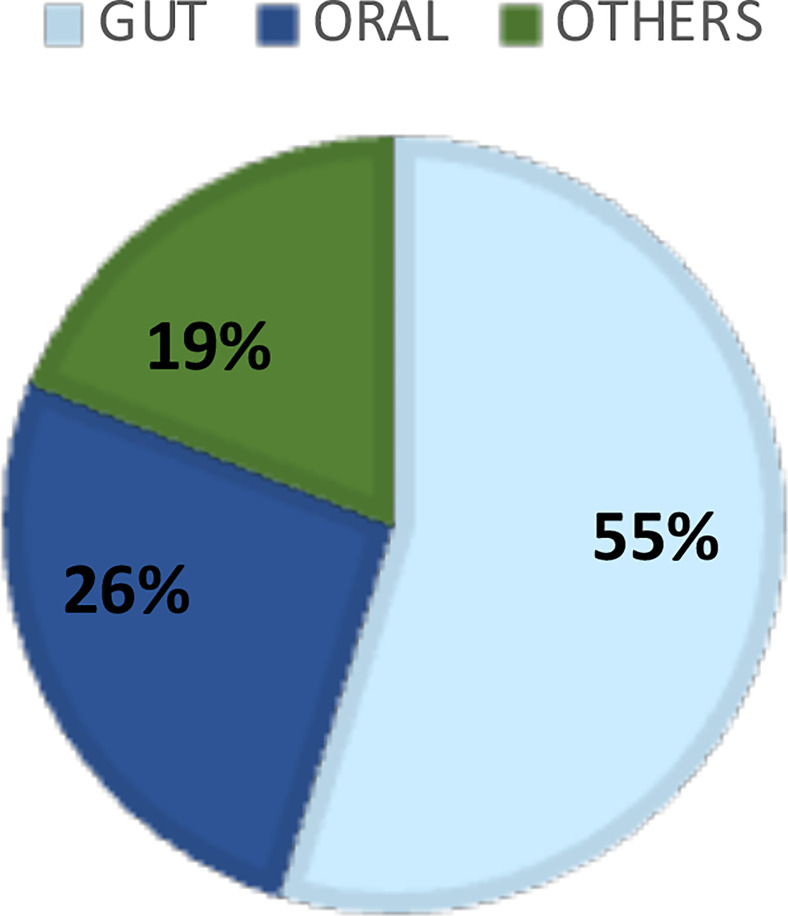
Distribution of the literature papers of low abundant organisms by sample’s sites.

The systematic literature search resulted in 826 manuscripts of which 67 were considered for full-text review after removing duplicates and applying the eligibility criteria. Following full text reading, 42 studies remained (**Figure 1**; **Table 1**). **Figure 2** shows the distribution of the papers by sampling site. Within them, the gastrointestinal tract and the oral cavity were the most studied ones. It may be due to the higher number of dysbiosis-related diseases or higher bacterial diversity in those sites, since only 10 out of the 42 articles exclusively analyzed samples from healthy individuals, and another 2 did not describe the status of health or disease, as they involved analysis of secondary data. The other sites included the vagina, respiratory system, skin, and blood. According to Hamady et al. (Hamady and Knight, 2009), the majority of microbiome studies describe the use of 16S rRNA gene sequencing for archaea and bacteria, and 18S rRNA gene sequencing for eukaryotes, which have limitations for the accurate identification to the species level.

**Table 1:** Qualitative Data Synthesis of the Included Studies (n = 42).

Reference	Sampling site	N	Platform of sequencing	Method of sequencing	Method of data analysis and bioinformatics	Proportion considered low abundant	Low-abundant microbiota
(Ai et al., 2017)	Oral	43	Illumina sequencing	Secondary data from metagenomics (Duran-Pinedo and Yost)	TagCleaner, PRINSEQ, Deconseq e FLASH, MetaPhlAn, GRAMMy, Network analysis	NA	*Porphyromonas gingivalis*, *Haemophilus haemolyticus*, *Prevotella melaninogenica*, and *Capnocytophaga ochracea* were considered potential keystones.
(Albert et al., 2015)	Vagina	310	454 GS Junior pyrosequencer	cpn60 PCR amplicon	Reads were mapped using Bowtie 2, microbial Profiling	4 species represented less than 0.3% of the overall reads mapped.	*Atopobium vaginae*, *Streptococcus devriesei*, *Lactobacillus acidophilus, Weissella viridescens.*
					Using Metagenomic Assembly pipeline (mPUMA)		
(Al-hebshi et al., 2016)	Oral	12	454 GS FLX pyrosequencer	16S rRNA (V1-V3)	Uchime, SILVA-HOMD database, ChimeraSlayer, BLASTN identity ≥98%	Together making up 0.77%	Saccharibacteria (TM7) and SR1.
(Balan et al., 2018)	Oral	24	Illumina MiSeq	16s rRNA (V4)	UPARSE (97%), Uchime, RDP Classifier v.2.2 against the Greengenes database, alignment at SILVA 108, Identification of keystone species was done using the CytoHubba plugin.	>0.5%<5.28% (keystones identification)	*Porphyromonas gingivalis* (2.22%),
							*Treponema denticola* (1.10%) and *Fretibacterium* sp. OT 361
							(0.67%) in supragingival plaque;
							*Prevotella_intermedia* (0.56%) in the saliva;
							*Porphyromonas endodontalis* (plaque 0.89%; saliva 0.91%).
(Brawner et al., 2017)	Stomach	86	Illumina platform	16S rRNA (V4)	UCLUST (100%), filter >10 reads, RDB classifier, Multiple sequence, alignment with PyNAST.	OTUs <1% were not analysed.	*H. pylori*-infected children harboured significantly reduced proportions of three bacterial classes (Actinobacteria, Bacilli, and Gammaproteobacteria), three orders (Pseudomonadales, Actinomycetales, and Lactobacillales) and four families (Streptococcaceae, Moraxellaceae, Actinomycetaceae, and Carneobacteriaceae) compared with fluids from non-infected children, but all with proportion >1%.
(Camelo-Castillo et al., 2015)	Oral	60	454 GS FLX pyrosequencer	16S rRNA	MG-RAST, Monthur, RDB classifier, BLASTN (>97%).	<1%	Desulfobulbus (especially D. propionicus) and Filifactor (F. alocis) with the periodontal inflammation severity, and a negative association of Anaeroglobus (especially A. geminatus) and TM7.
(Camelo-Castillo et al., 2019)	URT	56	454 GS FLX pyrosequencer	16S rRNA (V1-V4)	Prinseq, RDP database (80%), OUT>97% identify.	OTUs <0.1% were not analysed;	Corynebacterium, Neisseria, Actinomyces, or Rothia, among others, accounting for 9% of the reads.
						Low abundance at <1%.	
(Claussen et al., 2017)	Gut	822	454 GS FLX pyrosequencer	16S rRNA (V1-V2)	Entropy Shifts of Abundance Vectors under Boolean Operations (ESABO).	0.1%-0.4%	At phylum level: Chlorobi, Chloroflexi, Deferribacteres, Deinococcus-Thermus, Gemmatimonadetes, OP10, Planctomycetes, Thermodesulfobacteria, WS3.
(Dame-Teixeira et al., 2020)	Oral		Ion PGM	16S rRNA	Prinseq, USEARCH, UCLUST (97%); RDP, SILVA 132.	≤0.035%	Thaumarchaeota.
(Das et al., 2018)	Gut	84	454 GS FLX pyrosequencer	16S rRNA (V1-V5)	UCLUST (97%); RDP; SILVA.	0.01%-0.05% in at least 50% of the samples.	Phylum (Verrucomicrobia, Tenericutes and Fusobacteria);
							Class (Verrucomicrobia, Mollicutes and Fusobacteria);
							Order (Verrucomicrobiales, Bifidobacteriales, Desulfovibrionales, Anaeroplasmatales, Fusobacteriales, Rhizobiales, and Caulobacterales).
(de Goffau et al., 2013)	Gut	18	454 GS FLX pyrosequencer	16S rRNA (V1-V3)	RDP classifier, SILVA	OTUs <0.005% were not analysed;	Low abundance of Bifidobacteria and butyrate-producing species in children with β-cell autoimmunity.
						Low abundance at <12%.	
(Dobbler et al., 2017)	Gut	132	PGM Ion Torrent;	16s rRNA (V4);	BMP Operating System (BMPOS), UPARSE, UCLUST (97%) method against the Greengenes 13.5 database	>0.5 (16S rRNA)	Low abundance of *Lactobacillus* sp. in Necrotizing Enterocolitis (NEC);
			Oxford Nanopore MinION.	Metagenomics.		>0.38% (metagenomics)	4 day of life (without NEC) = Firmicutes (13.14%) and Actinobacteria (2.47%);
							5-7 day of life (without NEC) = Bacteroidetes (13.47%) and Actinobacteria (0.54%);
							Fungi (*Saccharomyceta* class) = 0.38%, no virus or archaea detected.
(Feng et al., 2015)	Ascites	7	Illumina Miseq	16S rDNA (v3)	BLAST NCBI (98.5% similarity);	NA	Cyanothece, Bacillus, Streptococcus; Salmonella, Pantoea, Cupriavidus; Rothia, Faecalibacterium, Acinetobacter.
			Illumina Hiseq	Whole Genome Sequencing (WGS)	NCBI mega-blast (90% identity).		The WGS approach was better at identifying microbes with a low abundance.
(Fisher and Mehta, 2014)	Gut	NA	NA	Secondary data from metagenomics (Caporaso et al.)	Data were obtained from the MGRAST database;	NA	*Bacteroides fragilis* and *Bacteroides stercosis* act as keystone species.
					Learning Interactions from MIcrobial Time Series (LIMITS).		
(Ghannoum et al., 2010)	Oral	20	454 GS FLX pyrosequencer	ITS1F-ITS4A (mycobiome)	BLAST Genbank (98%), Fungal ITS sequences were compared with the Assembling Fungal Tree of Life (AFTOL).	OTUs <1% were not analysed.	74 fungi genera detected (7 in high abundance);
							Authors declare that low-abundance genera may represent environmental fungi present in the oral cavity and could simply be spores inhaled from the air or material ingested with food.
(Hauser et al., 2015)	URT	54	454 GS FLX pyrosequencer	16S rRNA (V1-V3)	Uchime, BLAST SILVA, 111NR (95%)	OTUs <1% were not analysed.	20 minor bacterial species in one subject with completely negative culture;
							Low-abundance taxa were detected in 4.5% of cultures.
(Heisel et al., 2015)	Gut	11	Illumina MiSeq	ITS2 of the 18S rDNA fungal locus	UCLUST, USEARCH, alignment using MUSCLE, Mothur (hash.txt and fungalITSdatabaseID)	Present at <1.5% mean abundance across all samples.	*Candida krusei* and *Candida parapsilosis*.
				Validation with qPCR			
(Iebba et al., 2018)	Gut, blood	60	Illumina MiSeq	16S rRNA (V3-V4)	Python v.2.7.11, Mothur v.1.38.1, SILVA v.1.1961	≥0.5%	*Gemmiger formicilis, Oscillibacter ruminantium, Roseburia faecis and Faecalibacterium prausnitzii* were significantly higher in the controls than in cirrhotic patients, being classified as keystone species.
(Kang et al., 2017)	Gut	1463	Illumina MiSeq	16S rRNA (V3-V4)	USERCH 6.1 within the QIIME (97% similarity).	OTUs <0.005% were not analysed;	Unclassified Clostridiales (associated with the group with focal or intense FDG uptake in the intestine).
						Low abundance = 4.1%.	
(Kowalska-Duplaga et al., 2019)	Gut	82	Illumina MiSeq	16S rRNA (V3-V4)	QIIME2, DADA2, Greengenes database (99% similarity).	Low abundance in Crohn’s disease: 0.67%, 0.27%, 0.49%, 3.89%, 0.62%, and 0.35%, respectively.	Butyrate-producing bacteria, including Bifidobacterium (*B. adolescentis*), Roseburia (*R. faecis*), Faecalibacterium (*F. prausnitzii*), Gemmiger (*G. formicilis*), Ruminococcus (*R. bromii*) and Veillonellaceae (Dialister).
(Lau et al., 2016)	Gut	5	Illumina MiSeq	16S rRNA (v3)	Cutadapt, PANDAseq, AbundantOTU, QIIME, Greengenes database (97% similarity).	OTUs <0.01% were not analysed;	Uncultured OTUs were of low abundance (<0.8% relative abundance) in the culture-independent sequencing;
						Low abundance = <1%.	12 OTUs with relative abundances >0.1% were not cultured from the donor samples and included Cyanobacteria, Clostridia, Mollicutes, and Bacteroidetes.
(Li et al., 2013)	Oral, skin, distal gut, and vaginal	200	454 GS FLX pyrosequencer	16S rRNA	Taxonomic Variance, Binomial Distribution qualify Presence and Absence, Ubiquity vs. Abundance (Ub-Ab) Plots, Ubiquity-Ubiquity Plots (U-U Plots), HMP Consortium.	OTUs <0.01%/90% ubiquity were not analysed;	Buccal mucosa (Coriobacteriaceae Atopobium, Prevotellaceae unclassified, Bacilli unclassified, Lachnospiraceae Catonella).
						Definition of low abundance <1% (minor core taxa).	Hard palate (Clostridiales Family XIII. Mogibacterium, Lachnospiraceae Catonella).
							Keratinized gingiva (Bacilli unclassified).
							Palatine tonsils (Clostridiales Family XIII. Mogibacterium, Firmicutes unclassified).
							Saliva (Actinomycetales unclassified, Porphyromonadaceae Tannerella, Neisseriaceae Kingella).
							Subgingival plaque (Firmicutes unclassified).
							Supragingival plaque (Betaproteobacteria unclassified).
							Throat (Clostridiales Family XIII. Mogibacterium, Firmicutes unclassified). Tongue dorsum
							(Actinomycetales unclassified, Bacilli unclassified, Peptostreptococcaceae Peptostreptococcus).
							Anterior nares (Pseudomonadaceae Pseudomonas).
							Stool (Streptococcaceae Streptococcus).
(Li et al., 2019)	Oral	35	Illumina MiSeq	16 rRNA and ITS2	UPARSE (>97% identity), RDB classifier, UNITE database.	Core mycobiome: OTUs <0.1% were not analysed;	Keystone fungal genera (Bovista, Erysiphe, Psathyrella, etc.)
						Key oral fungal microbiota: OTUs with frequencies of at least 50% and relative abundances of ≥0.5% were analysed.	
(Ling et al., 2010)	Vaginal	100	454 GS FLX pyrosequencer	16s rRNA (V3)	MOTHUR (versão 1.5.0), RDP Classifier (80%), MEGA.	0.1-1.0% of total sequences.	Chloroflexi, Tenericutes, Proteobacteria and candidate division TM7;
							Mobiluncus.in low abundance (not described the %).
(Liu et al., 2019)	Gut	119	Illumina MiSeq	16S rRNA (V4-V5)	BIPES pipeline, AUCHIME, QIIME (1.9.1) USEARCH, PyNAST, Greengenes database, RDP Classifier.	OTUs with median in any group <0.3% were not analysed.	*Collinsella aerofaciens* and *P. copri* is a possible keystones for cardiac valve calcification and coronary artery disease.
(Nakayama et al., 2017)	Gut	43	454 GS FLX pyrosequencer	16S rRNA (V6-V8)	QIIME, USEARCH (97% identity), RDP classifier.	<1%.	Prevotellaceae in one of the groups of children.
(Nash et al., 2017)	Gut	147	Illumina MiSeq	16S rRNA (V3-V5)	USEARCH, UCHIME, NCBI GenBank Plant (including fungi) and Environmental databases, SILVA (bacteria), UPARSE, DIAMOND (metagenomics).	–	Mycobiome is relatively low abundant;
				18S rRNA (ITS2)			ITS2 sequencing provided greater resolution of the relatively low abundance mycobiome constituents.
				Metagenomics(fungi)			
(Ozkan et al., 2019)	Skin ocular	104	Illumina MiSeq	16S rRNA (V4)	UNOISE, USEARCH, Silva 128.	OTUs <1% across all samples were not analysed.	*Corynebacterium, Staphylococcus* in some sites.
(Rocas et al., 2016)	Oral	10	Illumina MiSeq	16s rRNA (V4)	Mothur v.1.36.1, Silva, UCHIME, RDP classifier (80%)	<0.1% not shown	Phylum level: Tenericutes, Synergistes
							Genus level: Megasphaera, Hawardela, Slakia, Filifactor, Parviromonas, Tannarella, Scardovia, others.
(Sakwinska et al., 2016)	Breast milk	90	Illumina MiSeq	16s rRNA (V4);	Mothur, Silva, RDP classifier (80%).	0.03%-0.5%.	Bifidobacteria and lactobacilli in low abundance in few samples.
				Confirmation by qPCR.			
(Simón-Soro et al., 2014)	Oral	13	454 GS FLX pyrosequencer	16S rRNA	Uchime, assigned to Ribosomal Database Project with 97% identity; RDP pyrosequencing pipeline; BLASTN>99%	0.02%- 1%	Tannerella, Olsenella, Filifactor, and Treponema (dentin carious lesions);
							*Streptococcus mutans* (enamel and dentin carious lesions);
							*Porphiromonas (*enamel carious lesions).
(Singh and Manning, 2016)	Gut	200	454 GS FLX pyrosequencer	16S rRNA (V3-V5)	QUIIME, USEARCH, Greengenes database,	No cuttoff defined in the methods, but OTUs with 0.03% were described.	*Bacterioides;*
							*Prevotella* in the group >70 years-old.
(Son et al., 2015)	Gut	59	Illumina MiSeq	16S rRNA (V1-V2 and V1-V3)	Uchime, Silva.	OTUs with a maximal relative abundance <0.0001 and with a prevalence <0.01 were culled;	Cyanobacterial, Chloroplast, Firmicutes, Asteroleplasma, Proteobacterial, Thalassospira, Burkholderia, Comanonadaceae, Bacteroidetes, Prevolellaceae, Actinobacteria, Mobiluncus, Sutterella, Bacteriodetes, Prevotella, Fusobacteria, Fusobacteriales.
						Low abundance (at the genera level) threshold of significance FDR<0.1	
(Wan et al., 2018)	Gut	30	Illumina MiSeq	16S rRNA (V3-V4)	QIIME, Monthur.	No cuttoff defined in the methods, but OTUs with 0.12% were described;	A high abundance of Proteobacteria and Fusobacteria was observed in most septic shock patients, whereas low abundance was observed in healthy subjects.
						Low abundance described as 3.53%, 0.12%.	
(Wang et al., 2017)	Oral	41	PacBio RS II	16S rRNA (V1-V9)	Pacbio circular consensus sequencing, Mothur v.1.36.1, UCHIME, QIIME (97% similarity).	OTUs with a median relative abundance <0.01% were not analysed.	*Haemophilus *spp., *Neisseria *spp., *Rothia *sp. *A. aphrophilus*, *Bergeyella *sp. clone oral AK152, and *S. rubneri* were in low abundance in both the caries group and the transitional group after the 6 month follow-up.
(Zhang et al., 2018)	URT	98	Illumina MiSeq	16S rRNA	QIIME.	No cuttoff defined in the methods, but OTUs with 0.42% were described.	Streptococcus and Rothia (0.68%) keep low abundance in orofarynx microbiota of children ≤1 year old;
							Oropharynx: Atopobium, Moraxella (0.42, 0.51%).
(Wu et al., 2019)	Gut	59	Illumina HiSeq	Paired-end metagenomic sequencing.	MetaPhlAn2.	Relative abundance lower than 5 in the centenarians;	A lower relative abundance for Faecalibacterium (*Faecalibacterium prausnitzii*), Ruminococcus (*Ruminococcus* sp_5_1_39BFAA), Corprococcus, *Eubacterium rectale*, and Dorea was observed in the centenarians;
						Low-abundant genera were summed into one group to plot.	Description of the *Archaea* domain; *Methanobrevibacter* was enriched.
(Zakrzewski et al., 2019)	Colon	73	Illumina MiSeq	16S rRNA (V3-V4)	QUIIME, Greengenes database (97% identity), UCLUST, UCHIME.	NA	On family level (Ruminococcaceae and Christensenellaceae abundance lower in ileal Crohn’s disease group).
(Zeng et al., 2019)	Gut	141	Illumina HiSeq 2500	16S rRNA (V4)	QIIME, Greengenes database (97% identity), DADA2.	<0.1%	Low abundance of butyrate-producing bacteria (Lachnospiraceae, Ruminococcaceae, Faecalibacterium, Roseburia, Lachnospira, and Oscillospira) with a higher risk of stroke.
(Zhang et al., 2015)	Antrum, proximal body and fundus	27	Illumina HiSeq 2500	Whole genome sequencing, confirmed by qPCR	PathSeq, Burrows-Wheeler Aligner, Bowtie2, PathoScope	No cuttoff defined in the methods, but OTUs with 0.001% were described.	The pipeline from the authors and Kraken identified high levels of *H. influenzae*
							(82.9 % and 75.4 %, respectively) as well as *P. acnes* (17.1 % and 24.6 %, respectively), whereas no bacteria were identified by MetaPhlAn;
							Main advantage of the approach from the authors over MetaPhlAn and Kraken is in samples with low levels of bacteria where the abundance of human DNA confounds bacteria detection.
(Zhang et al., 2019)	Gastric and esophageal sites	12	Illumina HiSeq 2500	Whole Genome Sequencing, confirmed by qPCR	Four different aligners: BWA, RepeatMasker, BLAST, MegaBlast.	NA	*H. pylori* in homogenization method, *Bifidobacterium* sp. and *Pantoea* sp. in lysis method.
(Zhu et al., 2018)	Gut	54	Illumina MiSeq	16s rRNA (V3-V4)	UPARSE, UCHIME, RDP classifier, Silva	53.36±21.44, 3.47±5.41, 1.93±2.71, 2.7±4.89, 1.04±1.92, respectively	Five bacterial families: Lachnospiraceae, Peptostreptococcaceae, Erysipelotrichaceae, Coriobacteriaceae, and Clostridiaceae_1 negatively associated with lipopolysaccharide level.

NA, Not available.

**Figure 3:**
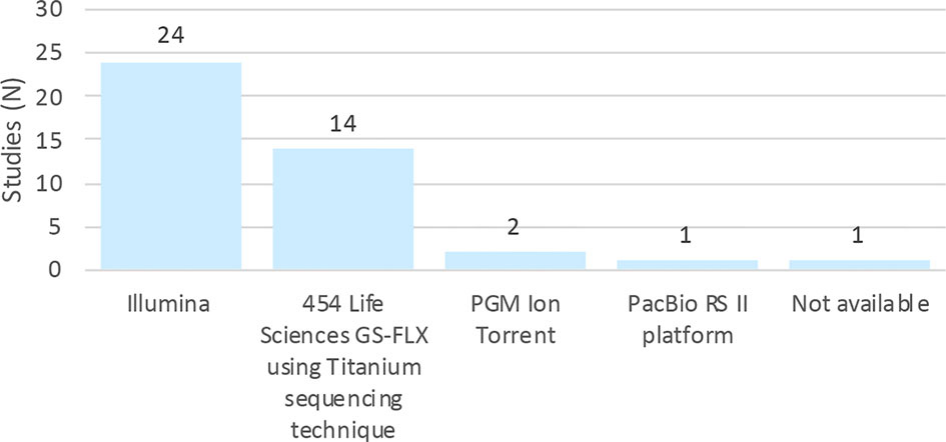
Distribution of studies by platforms of sequencing.

**Figure 3** shows the distribution of sequencing platforms used in the 42 selected articles. The most routinely used sequencing platforms were Illumina, followed by 454/Roche. Although these platforms are different in terms of biochemistry and in the way the matrix is generated, their workflows are conceptually similar (Shendure and Ji, 2008). A study of gut, mouth and skin samples from two subjects found that the composition of the gut and oral communities were not significantly dissimilar when either 454/Roche or Illumina (**Figure 3**) were used, albeit the communities of the skin were significantly different. This difference was attributed to bias associated with the primers (Caporaso et al., 2011).

### Low-Abundant Bacteria (LB)

Out of 42 articles, 20 were excluded from the summary of sample site-related low abundant bacterial species, because the data on microbial abundance were unavailable or no information on low abundance rate was provided. In the remaining 22 studies, low-abundant bacteria (LB) have been reported in the biofilm samples taken from 7 body sites (breast, gut, oral cavity, skin, stomach, upper respiratory tract (URT), and vagina). LB were determined and displayed as the relative abundance of a given operational taxonomic unit (OTU), relative to the total sequencing reads. In total, 4 studies reported LB at abundance levels between 5 and 20%, 6 studies reported between 1 and 5%, and 16 studies reported below 1%. Here we summarized the information of those LB detected at abundance levels below 1%. The information on bacterial phyla can be extracted from all 22 studies, hence it is possible to summarize the major phyla of LB per sample site.

**Table 2** summarizes how frequent a phylum was reported as LB (<1%) per site in the 22 studies. The frequency is indicated by the number of studies which have reported LB. In total, 6 different phyla have been reported as LB in more than 2 different studies or in more than 2 different body sites. Gut and oral cavity are the most examined body sites. Out of 6 different phyla, 5 phyla were reported in gut and 6 were reported in oral cavity. Actinobacteria and Firmicutes were the most frequently reported LB among various body sites. Actinobacteria has been found as LB in 6 different body sites. Firmicutes and Proteobacteria were found as LB in 5 different body sites. Compared to the gut, the oral cavity contains a site-specific LB phyla, Spirochaetes.

**Table 2:** Number of studies^a^ reported low abundant taxa (relative abundance <1%) at the level of phylum.

Phylum	Number of studies per site (n)							Total (n)	References
	Breast	Gut	Oral cavity	Skin	Stomach	URT	Vagina		
**Actinobacteria**	1	3	3	0	1	1	1	10	(Li et al., 2013; Simón-Soro et al., 2014; Albert et al., 2015; Son et al., 2015; Rocas et al., 2016; Sakwinska et al., 2016; Brawner et al., 2017; Das et al., 2018; Camelo-Castillo et al., 2019; Kowalska-Duplaga et al., 2019)
**Bacteroidetes**	0	2	4	0	0	0	0	6	(Li et al., 2013; Simón-Soro et al., 2014; Son et al., 2015; Rocas et al., 2016; Nakayama et al., 2017; Balan et al., 2018)
**Firmicutes**	1	4	3	0	1	0	1	10	(Li et al., 2013; Simón-Soro et al., 2014; Albert et al., 2015; Son et al., 2015; Rocas et al., 2016; Sakwinska et al., 2016; Brawner et al., 2017; Kowalska-Duplaga et al., 2019; Zeng et al., 2019)
Fusobacteria	0	3	1	0	0	0	0	4	(Son et al., 2015; Rocas et al., 2016; Das et al., 2018; Wan et al., 2018)
**Proteobacteria**	0	2	2	1	1	1	0	7	(Li et al., 2013; Son et al., 2015; Rocas et al., 2016; Brawner et al., 2017; Das et al., 2018; Wan et al., 2018; Camelo-Castillo et al., 2019)
Spirochaetes	0	0	2	0	0	0	0	2	(Simón-Soro et al., 2014; Rocas et al., 2016)

^a^the phylum reported by at least 2 different studies or found in at least 2 different body sites was included.

**Table 3** shows the bacterial taxa at the genus level within the major LB phyla (Actinobacteria, Bacteroidetes, Firmicutes and Proteobacteria) (<1% abundance). The oral cavity and gut were the most studied body sites, where a low-abundant genus was detected in more than two studies. The reported LB at the genus level in gut was generally different from those of the oral cavity. Only 3 LB genera have been found in both gut and oral cavity, namely, Bifidobacterium, Prevotella and Streptococcus. No LB genus can be reliably identified either in the gut or the oral cavity, since the listed genera were only reported by 1 or 2 studies, which may infer on the diversity of the LB in the human body, or could be biased by sequencing/analysis methods employed.

**Table 3:** Number of studies which reported low abundant taxa (relative abundance <1%) collected from gut and oral cavity.

Taxa identified		Number of studies per site (n)		References
Phylum	Genus	Gut	Oral cavity	
Actinobacteria	Actinomyces	—	1	(Simón-Soro et al., 2014)
	Atopobium	—	1	(Li et al., 2013)
	**Bifidobacterium**	1	2	(Simón-Soro et al., 2014; Rocas et al., 2016; Sakwinska et al., 2016; Kowalska-Duplaga et al., 2019)
	Mobiluncus	1	—	(Son et al., 2015)
	Olsenella	—	1	(Simón-Soro et al., 2014)
	Unclassified	1	1	(Li et al., 2013; Das et al., 2018)
Bacteroidetes	**Prevotella**	2	2	(Son et al., 2015; Rocas et al., 2016; Nakayama et al., 2017; Balan et al., 2018)
	Tannerella	—	2	(Li et al., 2013; Simón-Soro et al., 2014)
	Unclassified	1	1	(Li et al., 2013; Son et al., 2015)
Firmicutes	Catonella	—	1	(Li et al., 2013)
	Dialister	1	—	(Kowalska-Duplaga et al., 2019)
	Faecalibacterium	2	—	(Kowalska-Duplaga et al., 2019; Zeng et al., 2019)
	Filifactor	—	1	(Simón-Soro et al., 2014)
	Lachnospira	1	—	(Zeng et al., 2019)
	Oscillospira	1	—	(Zeng et al., 2019)
	Peptostreptococcus	—	1	(Li et al., 2013)
	Roseburia	2	—	(Kowalska-Duplaga et al., 2019; Zeng et al., 2019)
	Ruminococcus	1	—	(Kowalska-Duplaga et al., 2019)
	Staphylococcus	—	1	(Rocas et al., 2016)
	**Streptococcus**	1	1	(Li et al., 2013; Simón-Soro et al., 2014)
	Unclassified	2	1	(Li et al., 2013; Son et al., 2015; Zeng et al., 2019)
Proteobacteria	Burkholderia	1	—	(Son et al., 2015)
	Kingella	—	1	(Li et al., 2013)
	Ochrobactrum	—	1	(Rocas et al., 2016)
	Pseudomonas	—	1	(Rocas et al., 2016)
	Sutterella	1	—	(Son et al., 2015)
	Thalassospira	1	—	(Son et al., 2015)
	Unclassified	2	1	(Li et al., 2013; Son et al., 2015; Das et al., 2018)

—, indicates that the genus was not reported in this body site. The genera in bold are those identified in both gut and oral sites.

Actinobacteria were most often reported as a low-abundant phylum among all body sites. In the gut, Actinobacteria are relatively scarce, but have a high degree of ecological connection and are positively correlated with the diversity of the intestinal microbiome, playing an important role in the biodegradation of complex starch. It may be involved in the prevention of dysbiosis in patients with inflammatory bowel disease (Trosvik and de Muinck, 2015). When very abundant, Actinobacteria are associated with obesity (White et al., 2009). In the oral cavity, members of this phylum are part of the healthy microbiota and their abundance varies at each oral sites, however in dental plaque, for example, their abundance is less than 1% (Peterson et al., 2013; Palmer, 2014).

### Low-Abundance of Other Organisms

Archaea and fungi (eukaryotes) are usually reported in low abundance, however, this detection should be viewed with caution and further studies are always encouraged to validate and confirm the data. From the 42 selected articles, only 15 mentioned fungi and/or archaea, and from those only 4 (fungi) and 2 (archaea) showed data regarding the abundance of these domains. Ghannoum et al. (2010) described that low-abundance genera may be transient, and represent environmental fungi present in the oral cavity and could simply be spores inhaled from the air or material ingested with food (Ghannoum et al., 2010). They have shown several species not described before in the oral cavity. Heisel et al. showed *Candida krusei* and *Candida parapsilosis* in >1.5% mean abundance in all analysed faecal samples (Heisel et al., 2015). Wu et al., 2019, using shotgun metagenomics, identified methanogenic archaea within the core microbiota, enriched in individuals aged >100 years old (Wu et al., 2019). This technique may therefore be preferrable to 16S rRNA to identify this domain of microrganisms.

The low abundance related to these domains in other studies may be linked to the sample collection method, detection probe, pair of primers used, sequencing technique, and low number of sequences registered in current databases (Ghannoum et al., 2010; Heisel et al., 2015; Dame-Teixeira et al., 2020). Furthermore, the study of the microbial community through the use of 16S rRNA sequencing and shotgun metagenomic methods allows analysis of the composition and genetic capabilities of the microbiota, but not the particularities of the role of low abundance in the microbial community, and of microbial community interactions (Centanni et al., 2018). Microbial communities are complex and constantly changing in response to their environment, influenced by various factors such as diet, use of antibiotics, exposure to transient microorganisms. In this case, other OMICS techniques can be used to understand how microbes react to the environment, including metatranscriptomics, proteomics and metabolomics. Those approaches give a holistic view of the sample content, and a clearer idea of inter-domain interactions within the human microbiome.

### Bioinformatics and Data Analysis on Low-Abundant Organisms

Since 1977, DNA-sequencing technology has evolved at a fast pace, and is reshaping our understanding of biology (Srivastava, 2011). Next generation sequencing (NGS) was introduced for the first time in 2005, extending the previous advantages achieved by Sanger sequencing, and facilitated the increase in generated data, while decreasing the cost of sequencing (Buermans and Den Dunnen, 2014). NGS is marked by the construction of libraries, enabling massively parallel sequencing, which has been increasingly simplified, and a higher throughput compared to Sanger sequencing (Ekblom and Galindo, 2011; Muzzey et al., 2015).

Nevertheless, NGS has some limitations including issues with alignment of short read sequences, detection of artifacts and microbial contaminants present in samples, in addition to the presence of human nucleic acids in clinical samples, thus limiting the analytical sensitivity of microbial detection (Davis et al., 2018). One solution to this limitation was presented as the use of targeted sequencing of the 16S rRNA gene. This gene is now considered as a reference in microbial ecology studies. However, the use of 16S rRNA-based molecular methods do not allow for a high resolution of microbiota identification, because there are biases introduced into molecular community analysis by many factors, such as sample handling, DNA extraction, PCR and partial sequence of the 16S rRNA gene (ranging between the V1 and V4 regions) (Case et al., 2007). To reduce contamination with sequence artifacts or low accuracy of read alignment, some studies remove sequence reads attributed to low-abundance operational taxonomic units (OTUs) obtained by amplicon sequencing of the 16S rRNA gene. However, it is necessary to perform the analyses with caution, because sequence data associated with these low-abundant taxa may be biologically significant. Therefore, it may not be recommended to exclude these data even if the distinction between expected and unexpected sequences is not always straightforward (Lazarevic et al., 2016).

While microbiome studies generally describe the taxonomy, diversity and abundance of the highly abundant microbes, low-abundant species have been overlooked. Most studies included in this scoping review select a cut-off value at <1% for an organism to be considered low abundant, although some studies have reported OTUs representing 0.003% of the relative abundance (**Table 3**). The choice of such cut-off value were attributed to low read count and or other considerations such as technical artefacts, contaminations, and the presence of transient species. However, by excluding these OTUs from the analysis, the full richness and diversity of the microbiota is underestimated. Camelo-Castillo et al. (2019) stated that only the OTUs representing over 0.1% of the total sequences of each sample were considered for their analysis, as low-frequency reads, including singletons, are more likely to represent sequencing errors, contaminants, or transient organisms without a biological role at the niche under study. Although artifacts and errors are expected, important signals from low-abundant members of microbial community, including keystone organisms, may be lost due to the current technical limitations provided by this strategy. As affirmed before, low-abundant species can be responsible for major functions on the microbial community such as processing certain secondary metabolites. An example comprises organisms from the Archaea domain, that can be detected with 16S rRNA deep sequencing but in very low abundance. Those microrganisms, particularly the methanogens, play a unique role by using hydrogen to produce methane, modulating the environment and were previously described as keystone pathogens associated with periodontal diseases (Camelo-Castillo et al., 2019).

To overcome this limitation, an interesting approach was applied by Li et al (2019), that defined a core microbiome based on high ubiquity taxa in conjunction with a characteristic of high abundance such that the significance of both measurements can be made with a sufficient degree of confidence across and within samples. Using this approach, they were able to classify OTUs with low abundance (<1%) that were highly prevalent across the samples. The authors proposed that larger sample size and sequencing depth are necessary, so that the detection of low abundant taxa may be considered non-spurious across the donors (Li et al., 2019). We believe that defining the ubiquity of the low-abundant microrganisms is a good strategy that should be better explored. A clearer cut-off point to confirm the presence and importance of such species should urgently be defined (minimum values of the sample size, as well as the ubiquity).

Another approach was recommended by Liu et al. (2013), and based on single-read-based, instead of assembly-based classification which has a higher resolution for the characterization of the composition and structure of microbiota, especially for species in low abundance. Their composition and phylogeny-based algorithm uses the strategy of composition comparison, and is capable of classifying millions of very short reads relatively quickly (Liu et al., 2013). Zhang et al (2019) also described two DNA extraction methods (using prolonged lysis and homogenizing methods) which presented marked differences specifically to the low abundance genera (Zhang et al., 2019), and might represent an important improvement in the field.

Metagenomic studies produce high-throughput sequence data that attempt to classify the taxonomy and function of all microbial communities and are greatly affected by the presence of sequencing errors that may influence the estimation of taxonomic diversity (Keegan et al., 2012). There are noise and errors in the sequencing data that can be influenced by the type of platform used. In the studies included in this review, the most commonly used platform was Illumina. With this platform, when errors occur, they are predominantly substitution-type and the error percentage for most Illumina sequence reads is approximately 0.5% (1 error in 200 bases) (Mardis, 2013). The Ion Torrent PGM and 454 GS Junior platforms produced a higher error rate associated with homopolymers around 1.5 and 0.38 errors per 100 bases, respectively (Loman et al., 2012). All platforms are considered suitable for metagenomic sequencing, but no instrument can generate completely accurate data sets, each technology has advantages and disadvantages (Luo et al., 2012). The length of reads generated, sequencing depth and error rates may be taken into account when choosing the most appropriate platform to use. For example, longer reads as those provided by MiSeq (Illumina), Ion Torrent, PacBio and Oxford Nanopore Technologies, are important to consider when carrying out 16S rRNA metagenomics, or genome sequencing (Winand et al., 2020).

## Conclusion

There is currently no consensus in the literature on the classification of low-abundant organisms. Some studies have described such organisms being detected at less than 1% relative abundance, however, most studies use the same cutoff point (i.e. <1%) to exclude them, due to the risk of contamination or artifacts. This practice may compromise the identification of the true diversity of human microbiota. Domains other than *Bacteria* are neglected due to the cut-off, excluding OTUs with relative abundance lower than 0.1% or 1%. Representatives of Archaea, Fungi or Viruses are little explored. There is growing interest in developing new bioinformatics tools, such as single-read-based, instead of assembly-based, classification to obtain a higher resolution of the taxonomic analysis. Also, the ubiquity classification associated with the abundance could be a good strategy to identify the low-abundant microbiota. To achieve this, higher sequencing depths should be used in future microbiome investigations, as well as more holistic approaches including shotgun metagenomics should be employed to have a better view of the richness and diversity at play in health, disease and dysbiotic stages.

## Author Contributions

All authors listed have made a substantial, direct, and intellectual contribution to the work, and approved it for publication.

## Conflict of Interest

The authors declare that the research was conducted in the absence of any commercial or financial relationships that could be construed as a potential conflict of interest.
