# Supplementary material for: Low-Abundant Microorganisms: The Human Microbiome’s Dark Matter, a Scoping Review
Source: Front Cell Infect Microbiol. 2021 May 31;11:689197. doi: 10.3389/fcimb.2021.689197 (PMC8201079; doi:10.3389/fcimb.2021.689197)
Supplement: Supplementary file 1 [file Table_1.docx]

**Supplementary material (appendix describing the search process)**

**Appendix table 1 –** Search strategies according to different databases (7^th^ January, 2020)

| ***Databases*** | ***Search strategies*** | ***Results*** |
| --- | --- | --- |
| PubMed *via* MedLine | (("microbiota"[MeSH Terms] OR "microbiota"[All Fields]) OR ("microbiota"[MeSH Terms] OR "microbiota"[All Fields] OR "microbiotas"[All Fields]) OR "Microbial Community"[All Fields] OR "Community, Microbial"[All Fields] OR "Microbial Communities"[All Fields] OR "Microbial Community Composition"[All Fields] OR ("microbiota"[MeSH Terms] OR "microbiota"[All Fields] OR ("community"[All Fields] AND "composition"[All Fields] AND "microbial"[All Fields])) OR ("microbiota"[MeSH Terms] OR "microbiota"[All Fields] OR ("composition"[All Fields] AND "microbial"[All Fields] AND "community"[All Fields])) OR "Microbial Community Compositions"[All Fields] OR "Microbial Community Structure"[All Fields] OR ("microbiota"[MeSH Terms] OR "microbiota"[All Fields] OR ("community"[All Fields] AND "structure"[All Fields] AND "microbial"[All Fields])) OR "Microbial Community Structures"[All Fields] OR "Microbiome"[All Fields] OR "Microbiomes"[All Fields]) AND ("Human OR "[All Fields] AND ("microbiota"[MeSH Terms] OR "microbiota"[All Fields] OR ("human"[All Fields] AND "microbiome"[All Fields]) OR "human microbiome"[All Fields]) AND "OR "[All Fields] AND ("microbiota"[MeSH Terms] OR "microbiota"[All Fields] OR ("human"[All Fields] AND "microbiomes"[All Fields]) OR "human microbiomes"[All Fields]) AND "OR "[All Fields] AND ("microbiota"[MeSH Terms] OR "microbiota"[All Fields] OR ("microbiome"[All Fields] AND "human"[All Fields])) AND "OR "[All Fields] AND ("Gut"[Journal] OR "gut"[All Fields]) AND (("mouth"[MeSH Terms] OR "mouth"[All Fields] OR "oral"[All Fields]) AND ("dental health services"[MeSH Terms] OR ("dental"[All Fields] AND "health"[All Fields] AND "services"[All Fields]) OR "dental health services"[All Fields] OR "dental"[All Fields]) AND ("skin"[MeSH Terms] OR "skin"[All Fields]))  AND  (("minority groups"[MeSH Terms] OR ("minority"[All Fields] AND "groups"[All Fields]) OR "minority groups"[All Fields] OR "minority"[All Fields]) AND species[All Fields]) AND Title/Abstract[All Fields] AND (low[All Fields] AND abundance[All Fields]) AND Title/Abstract[All Fields] AND (("minority groups"[MeSH Terms] OR ("minority"[All Fields] AND "groups"[All Fields]) OR "minority groups"[All Fields] OR "minority"[All Fields]) AND phylum[Title/Abstract]) OR keystone[Title/Abstract]) | 245 |

| Cochrane | (Microbiota OR Microbiotas OR “Microbial Community” OR “Community, Microbial” OR “Microbial Communities” OR “Microbial Community Composition” OR “Community Composition, Microbial” OR “Composition, Microbial Community” OR “Microbial Community Compositions” OR “Microbial Community Structure” OR “Community Structure, Microbial” OR “Microbial Community Structures” OR “Microbiome” OR “Microbiomes” OR “Human Microbiome” OR “Human Microbiomes” OR “Microbiome, Human”) in Title, Abstract and Keyword AND (“minority species” OR "low abundance" OR “minority phylum” OR keystone) in Title, Abstract and Keyword | 14 trials |
| --- | --- | --- |
| Livivo | (Microbiota OR Microbiotas OR “Microbial Community” OR “Community, Microbial” OR “Microbial Communities” OR “Microbial Community Composition” OR “Community Composition, Microbial” OR “Composition, Microbial Community” OR “Microbial Community Compositions” OR “Microbial Community Structure” OR “Community Structure, Microbial” OR “Microbial Community Structures” OR “Microbiome” OR “Microbiomes” OR “Human Microbiome” OR “Human Microbiomes” OR “Microbiome, Human”) in Keyword AND (“minority species” OR "low abundance" OR “minority phylum” OR keystone) in Keyword - (removing the MedLine database) | 459 |
| Web of Science | TÓPICO: (((microbiota OR microbiotas OR "Microbial Community" OR "Community, Microbial" OR "Microbial Communities" OR "Microbial Community Composition" OR "Community Composition, Microbial" OR "Composition, Microbial Community" OR "Microbial Community Compositions" OR "Microbial Community Structure" OR "Community Structure, Microbial" OR "Microbial Community Structures" OR "Microbiome" OR "Microbiomes") AND (“Human” OR "Human Microbiome" OR "Human Microbiomes" OR "Microbiome, Human" OR “gut” OR oral OR dental OR skin))) AND TÓPICO: (("minority species" OR "low abundance" OR "minority phylum" OR keystone OR keystones)) | 285 |
| Scopus | TITLE-ABS-KEY((microbiota OR microbiotas OR "Microbial Community" OR "Community, Microbial" OR "Microbial Communities" OR "Microbial Community Composition" OR "Community Composition, Microbial" OR "Composition, Microbial Community" OR "Microbial Community Compositions" OR "Microbial Community Structure" OR "Community Structure, Microbial" OR "Microbial Community Structures" OR "Microbiome" OR "Microbiomes") AND (“Human” OR "Human Microbiome" OR "Human Microbiomes" OR "Microbiome, Human" OR “gut” OR oral OR dental OR skin)) AND TITLE-ABS-KEY("minority species" OR "low abundance" OR "minority phylum" OR keystone OR keystones) | 361 |
| ***Additional Grey Literature*** | ***Search strategies*** |  |
| Google Scholar | (“human Microbiota OR “Human Microbiome” OR “Microbiome” OR “Microbial Communities”) AND (“minority species” OR "low abundance" OR “minority phylum” OR keystone) | 13 |
| Open Grey | (Microbiota OR Microbiotas OR “Microbial Community” OR “Community, Microbial” OR “Microbial Communities” OR “Microbial Community Composition” OR “Community Composition, Microbial” OR “Composition, Microbial Community” OR “Microbial Community Compositions” OR “Microbial Community Structure” OR “Community Structure, Microbial” OR “Microbial Community Structures” OR “Microbiome” OR “Microbiomes” OR “Human Microbiome” OR “Human Microbiomes” OR “Microbiome, Human”) AND (“minority species” OR "low abundance" OR “minority phylum” OR keystone) | 2 |

**Appendix table 2.** Excluded studies and reasons for exclusion (n=25).

| **Author, year** | **Reason for exclusion** |
| --- | --- |
| Ana, E. et. al (2011) | 2 |
| Andersen, L. O. et. al (2016) | 2 |
| Cucchiara, S. et. al (2009) | 4 |
| Cummings, L. A. et. al (2016) | 5 |
| Diaz, P. I. (2012) | 4 |
| Dicksved et al. (2009) | 2 |
| El Kaoutari, A. et. al (2013) | 2 |
| Fu, B. C. et. al (2019) | 3 |
| Gotoh, A. et. al (2019) | 2 |
| Han, Y. et. al (2019) | 1 |
| Kawamura, Y. and Kamiya, Y. (2012) | 2 |
| Khemwong, T.et. al (2019) | 2 |
| Lawley, B. et. al (2017) | 2 |
| Li, M. and et. al (2018) | 2 |
| Liu, J. M. et. al (2013) | 6 |
| Lugli, G. A. et. al (2019) | 5 |
| Maldonado-Contreras, A. et. al (2011) | 2 |
| Mazzini, L. et. al (2017) | 3 |
| Reimers et al. (2016) | 3 |
| Rossen, N. G. et. al (2015) | 2 |
| Peterson et al. (2013) | 3 |
| Sousa, V. et. al (2017) | 3 |
| Trent, M. et. al (2019) | 3 |
| Tunney, M. M. et. al (2013) | 3 |
| Typpo, K. et. al (2016) | 7 |

1.Studies did not apply NGS methods (n=11); 2. Interventional designs (n=7); 3. Literature review (n=2); 4. Studies with no human sample (n= 2); 5. *In vitro* studies (n=1); 6. Studies written in non-Latin alphabet (n = 1); 7.Conference abstract (n=1)

**APPENDIX 2 REFERENCES**

Andersen, LO’Brien, et al. "Associations between common intestinal parasites and bacteria in humans as revealed by qPCR." European Journal of Clinical Microbiology & Infectious Diseases 35.9 (2016): 1427-1431.

Cucchiara, Salvatore, et al. "The microbiota in inflammatory bowel disease in different age groups." *Digestive Diseases* 27.3 (2009): 252-258.

Cummings, Lisa A., et al. "Clinical next generation sequencing outperforms standard microbiological culture for characterizing polymicrobial samples." *Clinical chemistry* 62.11 (2016): 1465-1473.

Diaz, Patricia I. "Microbial diversity and interactions in subgingival biofilm communities." *Periodontal disease*. Vol. 15. Karger Publishers, 2012. 17-40.

Dicksved, Johan, et al. "Molecular characterization of the stomach microbiota in patients with gastric cancer and in controls." Journal of medical microbiology 58.4 (2009): 509-516.

Duran-Pinedo, Ana E., et al. "Correlation network analysis applied to complex biofilm communities." *PloS one* 6.12 (2011): e28438.

El Kaoutari, Abdessamad, et al. "Development and validation of a microarray for the investigation of the CAZymes encoded by the human gut microbiome." *PLoS One* 8.12 (2013): e84033.

Fu, Benjamin C., et al. "Temporal variability and stability of the fecal microbiome: the multiethnic cohort study." *Cancer Epidemiology and Prevention Biomarkers* 28.1 (2019): 154-162.

Gotoh, Aina, Miriam Nozomi Ojima, and Takane Katayama. "Minority species influences microbiota formation: the role of Bifidobacterium with extracellular glycosidases in bifidus flora formation in breastfed infant guts." *Microbial biotechnology* 12.2 (2019): 259-264.

Kawamura, Yoshiaki, and Yasuyoshi Kamiya. "Metagenomic analysis permitting identification of the minority bacterial populations in the oral microbiota." *Journal of Oral Biosciences* 54.3 (2012): 132-137.

Khemwong, Thatawee, et al. "Fretibacterium sp. human oral taxon 360 is a novel biomarker for periodontitis screening in the Japanese population." *PloS one* 14.6 (2019): e0218266.

Lawley, Blair, et al. "Differentiation of Bifidobacterium longum subspecies longum and infantis by quantitative PCR using functional gene targets." *PeerJ* 5 (2017): e3375.

Li, Min, et al. "Identifying Keystone Species in the Microbial Community Based on Cross-Sectional Data." *Current Gene Therapy* 18.5 (2018): 296-306.

Liu, Jiemeng, et al. "Composition-based classification of short metagenomic sequences elucidates the landscapes of taxonomic and functional enrichment of microorganisms." *Nucleic acids research* 41.1 (2013): e3-e3.

Lugli, Gabriele Andrea, et al. "Uncovering bifidobacteria via targeted sequencing of the mammalian gut microbiota." *Microorganisms* 7.11 (2019): 535.

Maldonado-Contreras, Ana, et al. "Structure of the human gastric bacterial community in relation to Helicobacter pylori status." *The ISME journal* 5.4 (2011): 574-579.

Mazzini, Letizia, et al. "Potential role of gut microbiota in ALS pathogenesis and possible novel therapeutic strategies." *Journal of clinical gastroenterology* 52 (2018): S68-S70.

Peterson, Scott N., et al. "The dental plaque microbiome in health and disease." PloS one 8.3 (2013): e58487.

Reimers, Laura L., et al. "The cervicovaginal microbiota and its associations with human papillomavirus detection in HIV-infected and HIV-uninfected women." The Journal of infectious diseases 214.9 (2016): 1361-1369.

Rossen, Noortje G., et al. "The mucosa-associated microbiota of PSC patients is characterized by low diversity and low abundance of uncultured Clostridiales II." *Journal of Crohn's and Colitis* 9.4 (2015): 342-348.

Sousa, Vanessa, et al. "Peri‐implant and periodontal microbiome diversity in aggressive periodontitis patients: a pilot study." *Clinical oral implants research* 28.5 (2017): 558-570.

Trent, Maria, et al. "P595 Vaginal microbiota among adolescent and young adult women with pelvic inflammatory disease." (2019): A265-A265.

Tunney, Michael M., et al. "Lung microbiota and bacterial abundance in patients with bronchiectasis when clinically stable and during exacerbation." *American journal of respiratory and critical care medicine* 187.10 (2013): 1118-1126.

Typpo, Katri, et al. "Early Parenteral Nutrition Does Not Worsen Intestinal Barrier Function: A Pilot RCT: 8." *Journal of Parenteral and Enteral Nutrition* 40.1 (2016).

Yang, H. A. N., et al. "Analysis of uterine microbiota in abortion and non-pregnant female based on high-throughput sequencing." JOURNAL OF SHANGHAI JIAOTONG UNIVERSITY (MEDICAL SCIENCE) 39.2 (2019): 165.
